# Genetic Variation for Traits Related to Phosphorus Use Efficiency in *Lens* Species at the Seedling Stage

**DOI:** 10.3390/plants10122711

**Published:** 2021-12-10

**Authors:** Vinita Ramtekey, Ruchi Bansal, Muraleedhar S. Aski, Deepali Kothari, Akanksha Singh, Renu Pandey, Kuldeep Tripathi, Gyan P. Mishra, Shiv Kumar, Harsh Kumar Dikshit

**Affiliations:** 1 Division of Genetics, ICAR—Indian Agricultural Research Institute, New Delhi 110012, India; vinita14ramtekey@gmail.com (V.R.); murali2416@gmail.com (M.S.A.); deepalikothari1294@gmail.com (D.K.); 2 Department of Genetics and Plant Breeding, ICAR—Indian Institute of Seed Science, Mau 275103, India; 3 Division of Germplasm Evaluation, ICAR—National Bureau of Plant Genetic Resources, New Delhi 110012, India; Ruchi.Bansal@icar.gov.in (R.B.); Kuldeep.Tripathi@icar.gov.in (K.T.); 4 Amity Institute of Organic Agriculture, Amity University, Noida 201303, India; a_singh1388@yahoo.in; 5 Division of Plant Physiology, ICAR—Indian Agricultural Research Institute, New Delhi 110012, India; Renu.pandey@icar.gov.in; 6 Rabat-Institutes, ICARDA, B.P. 6299, Station Experiment, INRA-Quich, Rue Hafiane Cherkaoui Agdal, Rabat 10112, Morocco

**Keywords:** *Lens* species, phenotyping, root system architecture, phosphorus uptake, phosphorus utilization, phosphorus use efficiency

## Abstract

Phosphorus (P) is an essential, non-renewable resource critical for crop productivity across the world. P is immobile in nature and, therefore, the identification of novel genotypes with efficient P uptake and utilization under a low P environment is extremely important. This study was designed to characterize eighty genotypes of different *Lens* species for shoot and root traits at two contrasting levels of P. A significant reduction in primary root length (PRL), total surface area (TSA), total root tips (TRT), root forks (RF), total dry weight (TDW), root dry weight (RDW) and shoot dry weight (SDW) in response to P deficiency was recorded. A principal component analysis revealed that the TDW, SDW and RDW were significantly correlated to P uptake and utilization efficiency in lentils. Based on total dry weight (TDW) under low P, L4727, EC718309, EC714238, PL-97, EC718348, DPL15, PL06 and EC718332 were found promising. The characterization of different *Lens* species revealed species-specific variations for the studied traits. Cultivated lentils exhibited higher P uptake and utilization efficiency as compared to the wild forms. The study, based on four different techniques, identified EC714238 as the most P use-efficient genotype. The genotypes identified in this study can be utilized for developing mapping populations and deciphering the genetics for breeding lentil varieties suited for low P environments.

## 1. Introduction

The lentil (*Lens culinaris Medikus* ssp. *culinaris*) is a self-pollinated legume species with the genome size of 4063 Mbp/1C [1]. It is an ancient crop and its domestication dates back to the Neolithic Agricultural Revolution in the eastern Mediterranean during the 8th and 7th millennia BC [2]. Thereafter, the crop disseminated to Central Asia, the Nile Valley and Europe during the period of Neolithic agriculture. The lentil was also part of the Harappan crop assemblage (2250 to 1750 BC) in the Indian subcontinent [3]. The crop is cultivated for its protein-rich seeds and valuable straw in North America, South Asia, and the Mediterranean region. The global lentil production was 5.73 mtons during 2019 and Canada was the leading lentil producer followed by India [4].

Phosphorus (P) is a non-renewable and essential nutrient with limited global reserves [5]. The use of phosphate fertilizers has increased more than four times in the past five decades and is expected to reach 22–27 mton/year by 2050 [6]. Being an essential element, P is involved in the synthesis of DNA, RNA and membrane proteins and lipids. It acts as a signaling molecule and is associated with cellular phosphorylation events [7]. Plants absorb P from the soil mainly in the form of soluble inorganic P. In soil, P availability is low due to its fixation with calcium in acidic soils and iron/aluminum in alkaline soils [8]. Therefore, to ensure high yield, farmers apply excessive P fertilizers. However, plants utilize only up to 30% of the applied P and the rest is fixed in the soil or may cause eutrophication [9]. Improved P uptake and utilization is critical to reduce the cost of cultivation. The improvement of P use efficiency (PUE) becomes more important in legumes as legumes require more external P for growth and development as compared to other crops [10].

Efforts have been made by breeders to improve P uptake and utilization efficiency in different crops by targeting specific traits [11,12]. Root traits such as root dry weight (RDW), total root length (TRL), total surface area (TSA), total root volume (TRV), total root tips (TRT) and fork number (FN) involved in P uptake are of higher significance in P-deficient soils [13,14,15]. The phenotyping of black gram genotypes for root traits suggested that RDW is a potential trait for improving P uptake [16], whereas TRL, TSA, TRV, TRT and FN and carboxylate exudation efficiency were important in green grams [17,18]. Shoot dry matter, total dry matter, and shoot P concentration were associated with high PUE in soybeans [19], whereas long root hairs and a high shoot to root ratio were positively correlated to PUE in *Arabidopsis*. Limited reports are available on the genotypic diversity for PUE in legumes [20,21]. The cultivated lentil, *Lens culinaris* ssp. *Culinaris,* is native to near East and Central Asia. *Lens orientalis* is the probable progenitor [22] of the lentil. Later, this genus was divided [23] into four species including seven species/subspecies: *L. culinaris* ssp. *culinaris*, ssp. *orientalis* (Boiss.) Ponert, ssp. *tomentosus* (Ladizinsky) Fergusan, Maxted, van Slageren and Robertson, and ssp. *odemensis* (Ladizinsky) Fergusan, Maxted, van Slageren and Robertson, and *L. ervoides* (Brign.) Grande, *L. nigricans* (M.Bieb.) Godron and *L. lamottei* (Czefr). The different wild *Lens* species are distributed in the Mediterranean region. The present study is the first attempt to characterize the *Lens* genotypes’ germplasm under sufficient and low P conditions for the identification of PUE efficient lines and the identification of potential root and shoot traits associated with P uptake and utilization efficiency. The experiments were conducted with the objective of characterizing 85 *Lens* genotypes for the phenotypic variation of morphological root and shoot traits under sufficient and low P conditions, as well as to identify suitable genotypes for PupE and PUtE and phosphorus use efficient genotype(s).

## 2. Results

### 2.1. Variation in Root and Shoot Traits under SP and LP

The studied genotypes revealed variation for the root and shoot traits under SP and LP conditions. Plant growth was adversely affected in all the genotypes under low P conditions. Shoot growth was higher in SP conditions, whereas root traits showed genotype-dependent variation in response to LP (Table 1). All the studied traits registered wide variation in genotypic values under SP and LP conditions (Table 1). Most of the traits showed reduced expression under LP conditions. The percent reduction was 5.91% in PRL, 17.70% in TSA, 2.56% in ARD, 9.61% in TRV, 24.07% in TRT, 38.57% in RF, 39.62% in SDW, 36.21% in RDW, 38.74% in TDW, and 69.23% in PupE. Only the TRL and RSR increased by 14.05% and 7.58%, respectively, under LP compared to the SP level. PupE reduced drastically under LP conditions, with percent change of mean at −69.23 percent. PUtiE varied from 30.53 to 97.50% with a mean of 63.10% and CV% of 14.30%. The coefficient of variation (CV%) was high for TRV, TRT, SDW, TDW, RSR, PupE and under P-deficient conditions, whereas PRL, TRL, TSA, ARD, RF and RDW showed higher variation under P-rich conditions. (Table 1).

### 2.2. Estimation of Genetic Variance and Broad-Sense Heritability

The genetic variance components for different *Lens* species were significant for the shoot and root traits except for the ARD under contrasting P conditions (Table 2). Genotypic variance for the ARD under SP, genotype and treatment interactions were non-significant. The broad-sense heritability range for different traits was from 0.27 to 0.87 and 0.56 to 0.86 under SP and LP conditions, respectively. High heritability was recorded for studied traits in both SP and LP conditions, except for ARD in the SP condition (very low 0.27).

### 2.3. Genotypic Correlation among Root and Shoot Traits

The Pearson correlation analysis revealed the highly significant and positive correlations for most of the traits under SP (Table 3, upper diagonal) and LP (Table 3, lower diagonal). Significantly positive correlations were observed between TRL and PRL (r = 0.39), TRL and TSA (r = 0.91), TRL and TRV (r = 0.89), TRL and TRT (r = 0.75), TRL and RF (r = 0.82), TRL and RDW (r = 0.35) under LP.

TSA had a positive correlation with ARD, TRV, TRT, RF, RDW and PupE (r = 0.27, r = 0.91, r = 0.74, r = 0.81, r = 0.29 and r = 0.32, respectively), whereas TRV exhibited highly significant positive correlations with TRT, RF, SDW and RDW (r = 0.61, r = 0.76, r = 0.25, r = 0.32, respectively) under low P levels. TRT exhibited a positive correlation to RF, SDW, RDW, TDW and PupE (r = 0.61, r = 0.24, r = 0.29, r = 0.22 and r = 0.28, respectively) whereas RF exhibited a positive correlation with RDW (r = 0.37) under LP. Under SP conditions, TRL had a significantly positive correlation to TSA, TRV, TRT, and RF (r = 0.87, r = 0.84, r = 0.71 and r = 0.69, respectively) and TSA was positively correlated with TRV, TRT and RF (r = 0.88, r = 0.70, and r = 0.72, respectively). Some of the root traits exhibited negative correlation with SDW, RDW, TDW and RSR under both P regimes. The trait ARD was significantly correlated with PRL and TSA (r = 0.27 and r = 0.27, respectively) under low P environments and showed no correlation under sufficient P content. SDW was positively correlated to RDW and TDW (r = 0.67 and r = 0.94 at SP and r = 0.70 and r = 0.92 at LP). RDW was positively correlated with TDW (r = 0.84) at LP and at SP levels (r = 0.89). PupE had a significant and positive correlation with TRL, TSA, TRT, SDW, RDW and TDW (r = 0.37, r = 0.32, r = 0.28, r = 0.48, r = 0.41 and r = 50, respectively) whereas it was a non-significant correlation with PRL, ARD, TRV, and RSR under LP. PupE exhibited a significant correlation with TSA, TRV, RDW and TDW (r = 0.22, r = 0.24, r = 0.26 and r = 0.51, respectively) under SP.

### 2.4. Principal Component Analysis of Shoot and Root Traits

**Figure 1:**
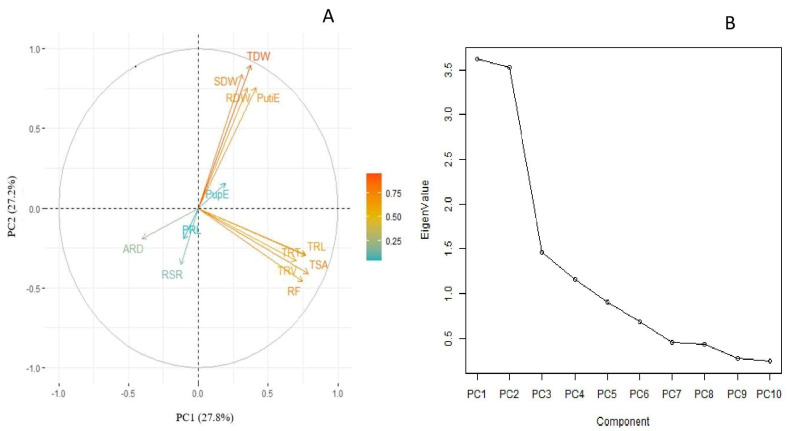
(**A**) Biplot and (**B**) scree plot using relative values of tested root and shoot traits of lentil germplasm lines under LP conditions. The arrow represents different root and shoot traits, whereas its length corresponds to the contribution of each trait for total variation. PRL, primary root length (cm); TRL, total root length (cm); TSA, total surface area (cm^3^); ARD, average root diameter (mm); TRV, total root volume (cm^3^); TRT, total root tips; RF, root fork; SDW, shoot dry weight (mg); RDW, root dry weight (mg); TDW, total dry weight (mg); RSR, root to shoot ratio (mg mg^−1^); PupE, P uptake efficiency (mg plant^−1^); PutiE, P utilization efficiency (%).

A principal component analysis (PCA) was carried out to identify the key traits contributing to responsiveness in lentils under P deficiency. PCA was analyzed via loading scores from the relative values of all traits under low P. The first two principal components explained 27.8% and 27.2% of the total variation for recorded traits under LP (Figure 1A). The biplot revealed that TRL, TRV, TRT, TSA and RF and their corelated traits such as RDW, SDW, and TDW are the key traits contributing to total variation. These root and shoot traits exhibited a significant correlation with PutiE and PupE. A scree plot between eigen values of factors and principal components depicted the maximum variation that resulted from PC1 to PC4. The eigen values from PC1 to PC2 are 3.61, 3.53, 1.46 and 1.16, respectively, whereas the percentage of explained variances from PC1 to PC2 are 27.8%, 27.2%, 11.3% and 8.9%, respectively (Figure 1B).

### 2.5. Genetic Variation in Lens sp.

**Figure 2:**
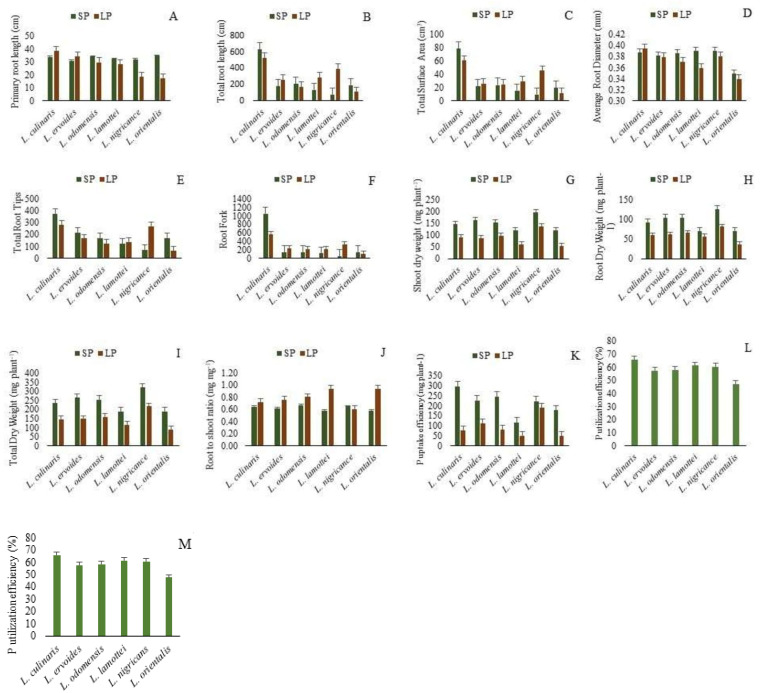
Performance of *Lens* sp. under sufficient phosphorus (SP) and low phosphorus (LP). (**A**) Primary root length (cm), (**B**) total root length (cm), (**C**) total surface area (cm^3^), (**D**) average root diameter (mm), (**E**) total root volume (cm^3^) (**F**) total root tips, (**G**) root fork, (**H**) shoot dry weight (mg plant^−1^), (**I**) root dry weight (mg plant^−1^), (**J**) total dry weight (mg plant^−1^), (**K**) root to shoot ratio (mg mg^−1^), (**L**) P uptake efficiency (mg plant^−1^), (**M**) P utilization efficiency (%); SP, sufficient P; LP, low P.

The genetic variation recorded for the studied traits in six different *Lens* species is presented in Figure 2. The highest PRL was recorded for *L. orientalis* under SP conditions. PRL recorded an increase in LP conditions in comparison to SP conditions for *L. culinaris* and *L. ervoides*. In the rest of the species, a relatively higher PRL was recorded under SP conditions (Figure 2A). A high TRL was recorded in *L. culinaris* under both LP and SP conditions, as compared to other *Lens* species (Figure 2B). Under LP conditions, higher TRL values were recorded for *L. ervoides, L. lamottei* and *L. nigricans* as compared to SP conditions in these species. The highest TSA was recorded for *L. culinaris* in both LP and SP conditions (Figure 2C). A high TSA in LP conditions was recorded in *L. ervoides*, *L. odemensis*, *L. lamottei* and *L. nigricans* as compared to SP conditions. A higher ARD was recorded for *L.nigricans*, *L. lamottie* and *L. culinaris*. The ARD increased under LP conditions as compared to SP conditions for *L. culinaris* (Figure 2D). *L.culinaris* exhibited a higher TRV in both LP and SP conditions, in comparison to other species. A high TRV was recorded for *L. nigricans* and *L. orientalis* in LP conditions as compared to SP conditions (Figure 2E). Maximum TRT values were recorded for *L. culinaris*. A higher TRT was recorded in LP conditions for *L. nigricans* and *L. lamotte* (Figure 2F). The RF increased in LP conditions over SP conditions for *L. ervoides*, *L. odemensis*, *L. lamottei* and *L. nigricans* (Figure 2G). Among the studied species *L. culinaris* exhibited a higher RF. The highest SDW, RDW and TDW were recorded for *L. nigricans* (Figure 2H–J). The SDW, RDW and TDW in LP conditions were lower than the SDW, RDW and TDW in SP conditions for all the studied species. The RSR values were higher in LP conditions in comparison to SP conditions in all studied species except *L. nigricans* (Figure 2K). The highest P uptake efficiency in LP conditions was recorded for *L. nigricans,* followed by *L.ervoides* (Figure 2L). The highest P utilization efficiency was recorded for *L. culinaris,* and an almost similar P utilization efficiency was recorded for *L. lamottei* and *L. nigricans* (Figure 2M).

### 2.6. Identification of Promising Genotypes for PupE and PutiE

Based on the TDW under low P levels, the top 10% of genotypes were identified as promising towards P deficiency (Table 4). The eight identified genotypes (L4727, EC718309, EC714238, PL-97, EC718348, DPL15, PL06 and EC718332) also had the top 10% for SDW, RDW, PupE and PUtiE. Among the eight identified genotypes, some of the common genotypes have shown better performance for SDW, RDW, PupE and PutiE. The genotypes EC718309, EC714238, EC718348 and PL06 belonged in the top 10% for TDW, SDW, and RDW. The genotypes EC718309 and EC718348 belonged in the top 10% for TDW, SDW, RDW and PupE. The genotypes PL06 and EC718332 belonged in the top 10% for TDW, SDW and PutiE. However, L4727 was in the top 10% for shoot dry weight and total dry weight, whereas DPL-15 belonged to the top 10% for traits TDW and RDW. The genotype EC718332 belonged in the top 10% for TDW, SDW, PupE and PutiE.

**Figure 3:**
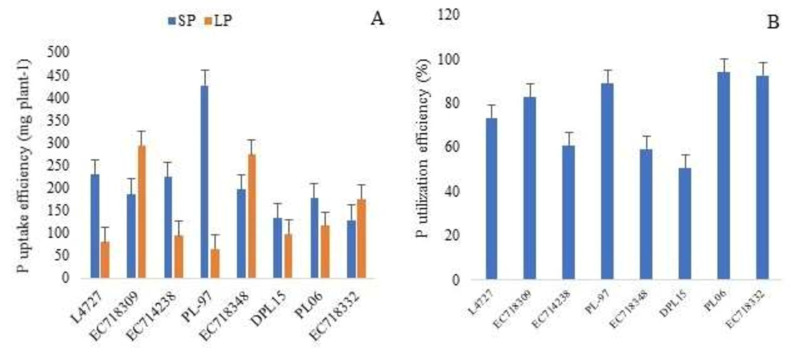
Performance of the top 10% of lentil genotypes selected based on total dry weight (**A**), P uptake efficiency (mg plant^−1^) (**B**), P utilization efficiency (%); SP, sufficient phosphorus; LP, low phosphorus.

PupE was highest for PL-97 (427 mg plant^−1^), followed by L4727 (229 mg plant^−1^), EC714238 (223 mg plant^−1^), EC718348 (195 mg plant^−1^), EC718309 (186 mg plant^−1^), PL06 (177 mg plant^−1^) and EC718332 (127 mg plant^−1^) under SP. PupE increased in some of the selected genotypes under LP compared to SP levels and was highest for EC718309 (295 mg plant^−1^), followed by EC718348 (273 mg plant^−1^), EC718332 (175 mg plant^−1^) and PL06 (115 mg plant^−1^) (Figure 3A). PUtiE was the highest for PL06 (94%), followed by EC718332 (93%), PL-97 (89%), EC718309 (83%), L4727 (73%), EC714238 (61%), EC718348 (59%) and DPL15 (51%) (Figure 3B). The results showed that these genotypes were highly efficient in uptake as well as the utilization of P under a limited P environment.

**Figure 4:**
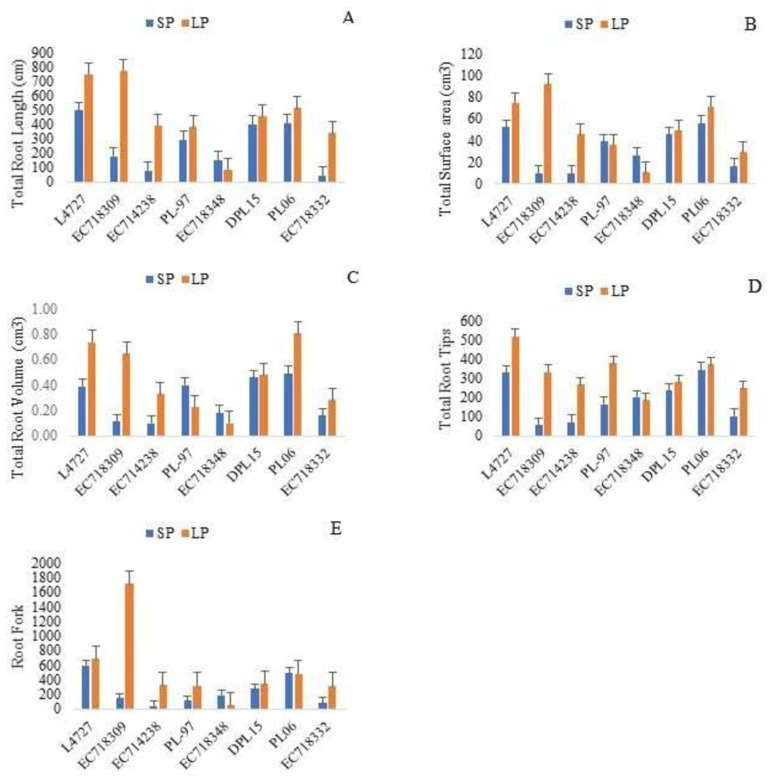
(**A**) Total root length (cm), (**B**) total surface area (cm^3^), (**C**) total root volume (cm^3^), (**D**) total root tips, (**E**) root fork in selected lentil genotypes.

Genotype, treatment and their interactions were significant for TRL, TSA, TRT, TRV and RF in the selected genotypes at a probability level 0.01 under two P levels (Table 5). As compared to SP levels, TRL, TRT, TSA, TRV and RF increased in L4727, EC718309, EC714238, DPL-15, PL06 and EC718332 in response to P deficiency, whereas they decreased non-significantly in the rest of the genotypes. PL-97 showed increased TRL, TRT, and RF but a reduction in TSA and TRV under LP. The genotype EC718332 exhibited reduction in all the root traits under LP (Figure 4).

The mean value of the studied traits of five genotypes selected based on PupE and PutiE under LP are presented in Table 6.

**Figure 5:**
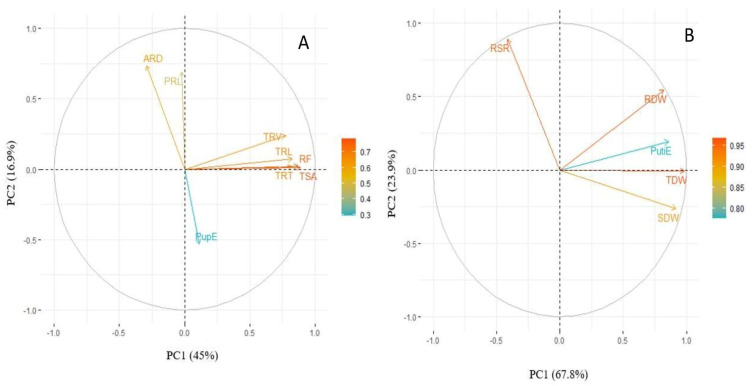
(**A**) Biplot PupE and (**B**) biplot PutiE using the relative values of tested root and shoot traits of lentil germplasm lines under LP conditions. The arrow represents different root and shoot traits, whereas its length corresponds to the contribution of each trait to the total variation. PRL, primary root length (cm); TRL, total root length (cm); TSA, total surface area (cm^3^); ARD, average root diameter (mm); TRV, total root volume (cm^3^); TRT, total root tips; RF, root fork; SDW, shoot dry weight (mg); RDW, root dry weight (mg); TDW, total dry weight (mg); RSR, root to shoot ratio (mg mg^−1^); PupE, P uptake efficiency (mg plant^−1^); PutiE, P utilization efficiency (%).

A principal component analysis (PCA) was carried out with the studied traits for PupE and PutiE under LP conditions. The first two principal components of the biplots explained 61.9% and 91.7% of the total variation, respectively (Figure 5A,B). The study of the PutiE biplot revealed that TRT, TRV, TRL, RF and TSA exhibited a positive correlation with PupE (Figure 5A). The biplot for PutiE revealed that RDW was positively correlated to PutiE. However, the TDW exhibited a meager contribution towards PutiE (Figure 5B).

### 2.7. Categorization of Lens Genotypes for PUE

Technique 1: The scoring of traits SDW, RDW, TDW, RSR, P percent, PupE, and PutiE under both the LP and SP revealed substantial variation among the 85 genotypes investigated (Table S2). The population was characterized for PUE based on the population mean and standard deviation of the studied traits. Under LP conditions, the genotype EC 714,238 scored highest (19 out of 20), followed by PL 06, WBL 81, SEHORE 74–3, MC 6, and EC 718,332 (18 out of 20). Under HP circumstances, the genotypes PL 05, EC 714238, and EC 718,339 scored highest (18 out of 20), followed by HUL 57, IPL 406, IC 268238, and L 4602 (17 out of 20). JL 1, IPL 406, L 4610, IG 129560, L 4650, EC 718464, EC 718312, EC 718281, and EC 718,282 (12 out of 20) in LP conditions and genotype L 4650 (11 out of 20) in HP conditions had the lowest scores among the 85 genotypes. By combining the scores at both P levels, EC 714,238 (37 out of 40) and EC 718,339 (34 out of 40) scored the highest for overall performance among the genotypes investigated. L 4717 and L 4650 had the lowest score (23 out of 30), indicating that they performed poorly for PUE among the genotypes.

**Figure 6:**
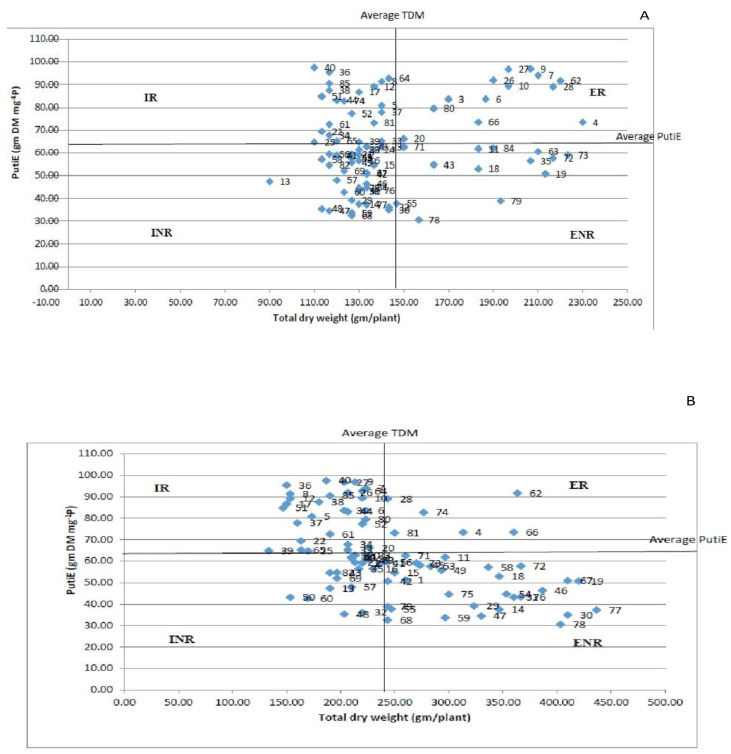
(**A**) Grouping of genotypes into four classes (IR, ER, ENR and INR) under HP conditions; (**B**) grouping of genotypes into four classes (IR, ER, ENR and INR) under LP conditions.

Technique 2: The genotypes were categorized into four groups based on TDM and PutiEin for both HP and LP regimes (Figure 6A,B). The genotypes PL06, DPL62, WBL-81, L4147, BM4, HUL-57 and EC718243 under LP conditions and genotypes L4727, EC718330 and EC718276 under HP conditions were classified in the ER group. Furthermore, the genotypes L4650, I-G-Y-50 and MC6 were grouped in the INR category under HP conditions. By contrast, under LP conditions, the genotypes IPL7103, EC 718464, KBL-104, EC 718,306 and EC 718,355 were categorized in the INR group. Interestingly, the genotypes PL-97, EC714238 and EC718339 were found in the ER group under both P conditions, whereas the genotypes EC718292, L4610, JL-1, EC 718351, EC718409 and EC718282 were categorized in the INR group under both P conditions. The genotypes IPL81, DPL15, EC718348 and EC718295 were categorized in the ENR group under both P conditions. The genotypes IC321808, JL-7, ILWL-118, L4649, P43120, IG134340, LH84-8, L4618, PL02 and L4076 were categorized in the IR group under both P conditions.

**Figure 7:**
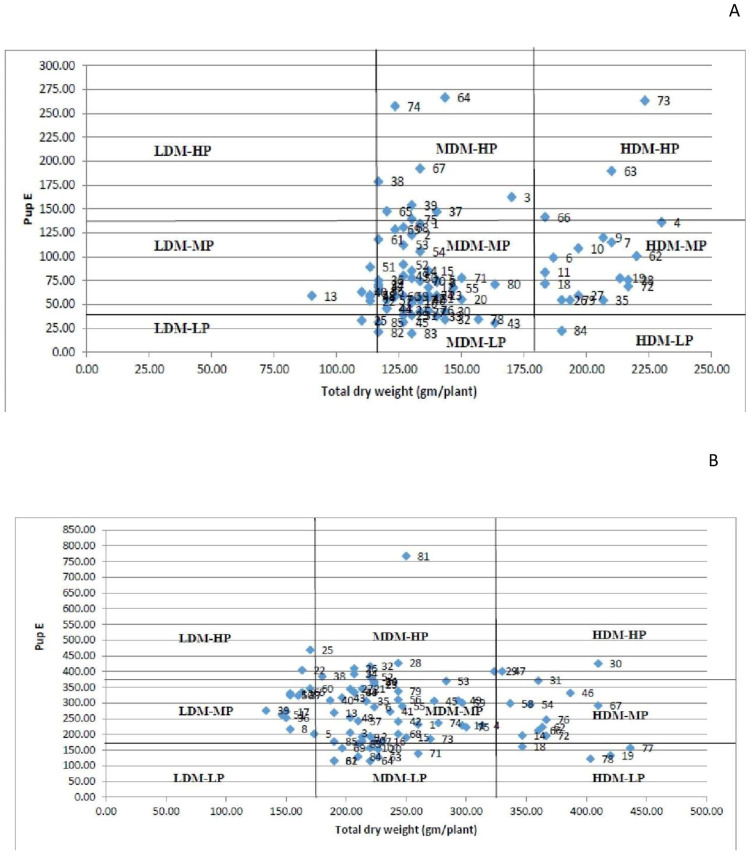
(**A**) Grouping of genotypes into nine classes under HP conditions; (**B**) grouping of genotypes into nine classes under LP conditions.

Technique 3: Under both P conditions, the categorization based on this technique results in the analysis of modest changes among genotypes. The HDM-HP group included the genotypes EC718309, EC718332, and EC718339 under LP conditions and PL-05, IPL7103, and IPL-406 under HP conditions (Figure 7A,B). Under LP conditions exclusively, the genotype PRECOZ was classified as LDM-LP. Under both circumstances, the genotype P43120 was classified as LDM-MP. In both HP and LP regimes, the genotypes EC718347, L4618, IC208352, and KBL-104 were classified as MDM-MP. Under both P circumstances, the majority of the genotypes were classified as MDM-MP. Under LP conditions, no genotypes were classified as LDM-HP, whereas under HP conditions, no genotypes were classified as LDM-LP. Surprisingly, genotypes EC718309 and PL-05 with a high TDM and PupE were classified as HDM-HP under both LP and HP conditions, whereas genotype EC718276 with a high TDM and PupE was classified as MDM-HP under HP conditions.

Technique 4: This non-graphical technique is based on the STS score tabulated based on seven P deficiency tolerance indices of 85 genotypes. Among the studied genotypes, the highest STS score was recorded by DPL15 (110.46) followed by EC 714,238 (87.86), whereas the lowest was recorded by P 43,120 (−60.47) followed by IG69568 (−58.16) (Table S3).

## 3. Discussion

The present study was designed to evaluate a panel of eighty-five diverse *Lens* genotypes for root and shoot traits at the seedling stage at contrasting P levels under controlled environments. The *Lens* genotypes were characterized for root and shoot traits in response to different P levels for the identification of superior *Lens* genotypes with significant P use. The most promising approach for developing a P use-efficient cultivar is by improving the key traits involved in the P uptake and utilization in the plant [24,25] Root architecture is instrumental to nutrient and water uptake, which has been poorly studied in lentils [26]. Significant phenotypic and genetic variations, moderate to high levels of heritability, and significant correlations among different traits were recorded at different P levels (Table 1, Table 2 and Table 3). This can be attributed to the diversity among and within the studied species for phosphorus uptake and utilization efficiency. These findings are in agreement with those reported in *Brassica* [27], common beans [28], maize [29] and green grams [18] under nutrient stress [18,30,31,32]. Significant genotypic variation was reported for root and shoot traits in rice [33,34], wheat [30], and mung beans [18] and for P use traits in mung beans [18]. Limited efforts have been made to study genetic diversity for phosphorus uptake and utilization efficiency in legumes.

A significant reduction in seedling growth traits (TDW, SDW, RDW) along with different root traits (PRL, TSA, ARD, TRV, TRT and RF) was recorded in response to P deficiency (Table 1). PRL, TSA, RV and root branching are important components of root architecture and play important roles in determining the rate of nutrient uptake. Although genotype-dependent variation was evident for different traits, most of the genotypes registered a significant reduction in response to P deficiency. In mung beans, P-efficient genotypes exhibited a better RSA, TRV and carbon exudation efficiency [17]. In rice, the RDW and RSR increased with a reduction in the SDW [26]. Root architectural plasticity was correlated to the RDW, root length density, and lateral roots in response to low P [35].

The Pearson correlation coefficient explained a highly significant correlation among most of the traits at the seedling stage under different P levels (Table 3). We noted significant correlation between the TRL and TSV, TRV, TRT and RF in lentils. Similarly, the TRL was positively correlated with the TSA and TRV in maize [36], with the TRT and RF in mung beans [18], and the SDW, RDW, and TDW under both SP and LP conditions. In the present study, the TDW was positively correlated with the RDW and SDW and negatively correlated to the RSR under low P levels (Table 3). The ARD showed a non-significant correlation to most of the traits in the present study as observed in chickpeas [37]. In contrast to our findings, the ARD was highly significant and negatively correlated with the TRT and RF under LP in mung beans and can be used as an important trait to differentiate nutrient availability [18]. A significant correlation among the root traits TRT, TRL, RSA, TRV, and ARD was reported in maize at the seedling stage [38]. 

The PCA identified the most contributing traits responsible for total variation as the TRL, TSA, TRT, RF, SDW, RDW, TDW, PutiE and PupE under LP (Figure 1). The present results are in accordance with previous studies, where P stress alters the TRL, SDW, TSA and TRT in mung beans [18] and the TRL and TSA in common beans [28]. We identified promising genotypes based on the TDW under P-deficient conditions (Table 4). The selected superior genotypes belonged to the top 10% for SDW, but few differed in ranking for TRL, RDW and SDW under SP and LP, which can be due to variation in the root, shoot traits and genotype x treatment interaction [39,40,41]. We observed that the SDW was positively correlated with the RDW and TDW under both P conditions. The RDW was positively correlated with the SDW under stress conditions in maize [42], whereas the TRL and RDW were significantly correlated with root traits under P and N stress conditions in maize [15,43]. It was suggested that the TDW, RDW, and SDW are potential phenotypic traits for the selection of lentil genotypes under P efficiency. The evaluation of soybean germplasm revealed moderate to higher values of heritability for most of the root and shoot traits [44,45]. Traits such as the TRL, TSA, TRV, TRT, RDW, and SDW contributed most to the genetic diversity and significantly correlated with P accumulation under SP and LP conditions [46,47]. This result was similar to the results of previous studies, which revealed that the traits TRL, TSA, RDW, and SDW are the major traits for selection of genotypes in maize under different stress conditions [36,42].

In the present study, we observed that genotypes with a high TDW and SDW under low P were also promising for PupE and PutiE (Table 4). PUtiE was also significantly correlated with TDW under different P regimes in rice [48]. Though the genotypes which had higher TDW along with RDW showed better performance for physiological P use. It can be attributed to the correlation between RDW and different root traits, which resulted in efficient P uptake in these genotypes. The increased root length was responsible for higher P uptake from low P supply in barley and sugar beet roots [49,50] whereas the TRT, RSA, TRL and root hair length were important in P uptake in other studies [51,52]. In rice, the genotypic variation in P uptake was due to the RDW and RSA under P deficiency [53]. The potential use of *L. ervoidis, L. nigricans* and *L. culinaris* ssp. *odemensis* for drought tolerance [54], and *L. culinaris* ssp. *orientalis* for cold tolerance [55] and salinity tolerance [56] has been investigated. The utility of wild *Lens* species for phosphorus uptake and utilization requires an investigation with a large number of genotypes of each species/subspecies. In this study a limited number of genotypes of wild *Lens* were investigated. The variations were recorded for different studied traits at LP and SP conditions in this study.

According to the classification approach reported earlier [33,57], lentil genotypes were classified into four categories based on P efficiency and responsiveness: ER, ENR, IR, and INR. Under both P circumstances, the most efficient genotypes, EC714238 and EC718339, were classified as ER. Under HP conditions, the genotypes EC 718,330 and EC 7188276, which were in the ER category under LP, were moved to the IR category. This emphasizes the significance of categorization at both high and low P levels. The genotypes classified as ER were well suited to soils with variable levels of P. The genotypes in the ENR group, on the other hand, could be successfully cultivated in P-depleted soils. The IR genotypes could be employed in a crossbreeding program to incorporate P-responsive characteristics. The genotypes of the INR category, on the other hand, have no part in the PUE enhancement program [58]. This method of categorization allows for the identification of genotypes suitable for a variety of growing conditions and P levels [59]. This strategy, however, is primarily dependent on the population mean. As a result, the difference between efficient and inefficient, responsive and nonresponsive kinds is relatively thin [58]. The genotypes EC 718,276 and PL 97 in the LP condition and NDL-1 and EC 718,287 in the HP condition, for example, were at the borderline between efficient and inefficient groups. As a result, genotypes with a minor deviation from the population mean are difficult to categorize as efficient or inefficient, or responsive or nonresponsive. As a result, this technique is ineffective for investigating and categorizing genotypes on a wide scale [57].

The genotypes were classified into nine groups (LDM-HP, LDM-MP, LDM-LP, MDM-HP, MDM-MP, MDM-LP, HDM-HP, HDM-MP, and HDM-LP) using a graph with the TDM and TPU on the x and y axes, respectively, in both HP and LP conditions [60]. By generating nine groups, this categorization method may discern tiny changes between genotypes [59]. This method, on the other hand, is more suited to categorizing genotypes at low P levels [57]. The efficient genotype EC714238 with high TPU and TDM was clustered in HDM-MP under both HP and LP conditions in this investigation. HDM-HP genotypes are efficient in P uptake, and their use for biomass production suggests the genotype’s ability to produce greater biomass under a variety of P regimes [30]. L 4717 was classified as MDM-MP in both LP and HP situations, whereas L 4650 was classified as LDM-MP in LP and MDM-MP in HP. Both P absorption and use in biomass production are inefficient in LDM-LP genotypes. The genotypes in the LDM-MP group are good at absorbing P but not so good at being used for biomass production.

As a result, the three-way categorization of genotypes (low, medium, and high) allows for the discovery of significant differences between the high and low groups while also providing the most room for medium type genotypes. Such differences explain genotype adaptation across a wide range of P regimes and provide the genetic foundation for PUE enhancement in breeding strategies. The categorization of genotypes using stress tolerance indices calculated from genotype dry mass under control and stress situations was reported earlier [32,61]. The P deficiency tolerance indices of all genotypes were computed using TDM under both HP and LP conditions in the current investigation. The susceptibility indices SSI, TI, and SI have a negative association with yield/biomass and are used to distinguish between stress-tolerant and susceptible genotypes [62], whereas the tolerance indices MPI, GMPI, and STI have a positive connection with yield/biomass and can be used to select genotypes with high average yield/biomass and stress tolerance [63].

The studied *Lens* genotypes exhibited significant genetic variability and heritability for different root and shoot traits at two contrasting levels of phosphorus. Most of the recorded parameters revealed a remarkable reduction in P deficient conditions. Wild *Lens* species exhibited low PupE and PutiE in comparison to cultivated species (Figure 2L,M). Genotypes EC718309, EC718348, EC718332 and PL06 were better in P uptake as well as in P utilization compared to other genotypes. The identified genotypes can be utilized for the development of mapping population for the identification of QTLs responsible for P uptake and utilization efficiency. The characterization of the studied genotypes using different techniques identified the genotype EC714238 as having the highest STS score, indicating that it was highly phosphorus use efficient under LP conditions. The genotype can be utilized in breeding programs and in genetic studies.

## 4. Materials and Methods

### 4.1. Plant Materials and Plant Growth Conditions

A diverse set of eighty-five *Lens* genotypes, including twenty-six cultivated varieties, twenty-eight advanced breeding lines and thirty-one wild species accessions, were used for studying root and shoot traits under sufficient phosphorus (SP) and low phosphorus (LP) conditions (Table S1). The seeds of wild *Lens* species were provided by ICARDA, Aleppo, Syria. The characterization of eighty-five *Lens* genotypes was carried out under hydroponic conditions at two levels of phosphorus, consisting of sufficient P (SP) and low P (LP). The experiment was conducted under a greenhouse at the National Initiative on Climate Resilient Agriculture (NICRA), a controlled environment facility of the Indian Agricultural Research Institute, New Delhi, India in 2019 (winter season). The growth conditions maintained throughout the study period were day and night temperatures of 25 and 16 °C, respectively, a photoperiod of 12 h, and the relative humidity at 85%. The seeds were surface sterilized with 0.1% (*w*/*v*) H_g_Cl_2_ for 3 min followed by double distilled water rinsing. The seeds of wild *Lens* species were scarified, wrapped in germination paper and kept in the dark for the germination. After the emergence of cotyledonary leaves, 8–10-day-old seedlings of uniform size were transferred to the Hoagland solution. The basal macronutrient solution consisted of 1 mM MgSO_4_, 0.92 mM K_2_SO_4_, 0.75 mM CaCl_2_.2H_2_O, 0.04 mM Fe-EDTA, 5 mM Urea, and a micronutrient combination of 2.4 μM H_3_BO_3_, 0.9 μM MnSO_4_, 0.6 μM ZnSO_4_, 0.62 μM CuSO_4_, and 0.6 μM Na_2_MoO_4_ [64]. Plastic trays (30 × 45 × 15 cm) with a capacity of 10 L basal nutrients were used for hydroponics. The 2” thick thermocol sheet was used to support the seedlings, which had holes at a distance of 5 × 5 cm to maintain row-to-row and plant-to-plant spacing. Fifteen genotypes with three replications were raised per container. The aquarium air pump was used to maintain air circulation in the medium. The nutrient solution was replaced on alternate days to avoid any microbial contamination. Using 1 M KOH or 1 M HCL, the pH of the nutrient solution was maintained at 6.0. The N:P ratio in hydroponic solution was 16N:1P in the SP condition and 1666 N:1P in the LP condition. The study was conducted with a series of P concentrations to select the sufficient and low P levels. The analysis of the observations on chlorophyll content, biomass and visual symptoms led to the selection of the sufficient (300 μM) and low (3 μM) P concentrations, and the phosphorous was supplemented in the form of KH_2_PO_4_. 

### 4.2. Trait Measurements

One-month-old seedlings were harvested to study the root and shoot traits raised under SP and LP conditions. The roots were separated from the shoots and were scanned using an Epson Perfect V700 Pro scanner (Seiko Epson, Suwa, Japan). The grayscale images in TIFF format were studied with WinRHIZO Pro 2016a software. The root system was spread in an acrylic tray, avoiding overlapping among them. The broken root segments were manually separated during the root scanning. Based on an image analysis of the root architecture, the traits measured were primary root length (PRL, cm), total root length (TRL, cm), total surface area (TSA, cm^2^), average root diameter (ARD, cm), total root volume (TRV, cm^3^), total root tips (TRT, cm), and root fork (RF). The shoots and roots of the plants were oven dried at 65 °C for 48 h to obtain the desired shoot dry weight (SDW, mg plant^−1^), root dry weight (RDW, mg plant^−1^), total dry weight (TDW, mg plant^−1^), and root to shoot ratio (RSR, mg mg^−1^).

### 4.3. Estimation of P Concentration

For the P estimation, 0.1 gm grounded samples of the studied genotypes were digested with a 10 mL di-acid mixture (HNO3: HClO4, 9:4) and a volume that was made up to 50 mL. It was filtered through Whatman No. 42 filter paper. The samples were run on an inductively coupled plasma optical emission spectrometer (ICP-OES; model 5110, Agilent Technologies (Santa Clara, CA, USA) which was calibrated using the standard for measuring the absorbance of the blue-colored phosphomolybdate complex at 660 nm [65]. The results of four replications were averaged and the P concentration was expressed as mg g^−1^ dry weight. The P uptake efficiency (PupE) and P utilization efficiency (PutiE) were calculated using the following formulas [60,66]:

PupE (mg plant^−1^) = P concentration (mg mg^−1^) × dry matter (mg plant^−1^)

PutiE (%) = TDW (LP)/TDW (SP) × 100

### 4.4. Estimation of Phosphorus Use Efficiency

The PUE was measured using four techniques. Technique 1 [67,68], the studied genotypes were classified (based on population mean and standard deviation) as efficient (>µ + SD), medium (µ + SD to µ − SD) and inefficient (<µ − SD). Technique 2 [33,34], classified *Lens* genotypes in four groups: efficient responsive (ER), efficient non-responsive (ENR), inefficient and non-responsive (INR) and inefficient but responsive (IR). Technique 3 [30], grouped *Lens* genotypes by plotting TPU and TDM on the y-axis and x-axis, respectively, in nine groups: high dry mass-high P (HDM-HP), high dry mass-medium P (HDM-MP), high dry mass-low P (HDM-LP), medium dry mass-high P (MDM-HP), medium dry mass-medium P (MDM-MP), medium-dry mass-low P (MDM-LP), low dry mass–high P (LDM-HP), low dry mass-medium P (LDM-MP) and low dry mass-low P (LDM-LP). Technique 4 [31,32], utilized stress tolerance score for characterizing the genotypes for PUE

Stress tolerance score (STC) = SSI + MPI + GMPI + HMI + STI + TI + SI

where stress susceptibility index (SSI) = (1 − *T*/*C*)/(1 − *xT*/*xC*), mean productivity index (MPI) = (*C* + *T*)/2, geometric mean productivity index (GMPI) = √C × T√C, harmonic mean index (HMI) = 2 (*C* × *T*)/(*C* + *T*), stress tolerance index (STI) = (*C* × *T*)/(*xC*)^2^, tolerance index (TI) = *C* − *T* and stress index (SI) = *T*/ where *C* represents the total dry mass under control conditions and *T* represents the total dry mass under treatment conditions. *xC* and *xT* represent the average total dry mass (TDM) of all studied genotypes under SP (control) and LP (treatment) conditions, respectively. 

### 4.5. Statistical Analysis

The STAR (Statistical Tool for Agricultural Research) 2.1.0 software was used to estimate the coefficient of variation, variance components to predict genotypic values and the Pearson correlation coefficients under SP and LP conditions [69].

The percent change in response to P stress was calculated as follows: {[(SP − LP)/SP] × 100}. The broad-sense heritability was estimated based on expected mean squares for each trait under both SP and LP and with combined analysis as follows [70]:

Broad-sense heritability H= σ2G/(σ2 G+σ2 e/r)(1)

and

Combined heritability H_com_ = σ2G/(σ2G+(σ2GE)/e) + (σ2e)/re))

where σ2G, σ2GE and σ2 e are genotypic variance, variance due to genotype × P level interaction, and error variance, respectively; ‘*r*’ is the replication.

All the measured traits were subjected to principal component analysis (PCA) to identify the common trends of the multidimensional data sets. The principal component analysis (PCA) was performed for different root and shoot traits by using relative values under LP with the help of an R software package “FactoMineR” to detect the most contributing traits [71].

## Figures and Tables

**Table 1:** Estimates of ranges and means, coefficient of variation (CV%), and percentage change in means for root and shoot traits under SP and LP in lentils.

Traits	Max		Min		Mean		CV%		% Change in Mean
	SP	LP	SP	LP	SP	LP	SP	LP	
PRL	50.00	46.83	19.50	17.33	35.72	33.61	9.11	5.86	−5.91
TRL	1211.40	1380.41	43.42	80.44	366.79	418.34	10.68	8.61	14.05
TSA	171.87	133.05	9.45	9.08	59.02	48.57	10.26	9.62	−17.70
ARD	0.47	0.46	0.34	0.33	0.39	0.38	7.17	6.97	−2.56
TRV	1.80	1.35	0.10	0.08	0.52	0.47	11.14	13.72	−9.61
TRT	873.67	640.33	55.00	42.40	309.70	235.14	10.10	13.14	−24.07
RF	5630.67	1830.33	31.67	44.33	725.67	445.80	13.71	10.41	−38.57
SDW	260.00	163.33	78.89	55.56	148.65	89.76	12.60	13.61	−39.62
RDW	173.33	88.89	56.67	43.33	95.94	61.20	11.28	8.67	−36.21
TDW	424.44	235.56	151.11	103.33	244.59	149.84	11.35	10.26	−38.74
RSR	0.99	1.14	0.43	0.40	0.66	0.71	9.86	10.73	7.58
PupE	768.01	266.49	115.65	19.50	275.65	84.80	11.03	14.20	−69.23
PutiE	97.50		30.53		63.10		14.30		

SP, sufficient phosphorus; LP, low phosphorus; PRL, primary root length (cm); TRL, total root length (cm); TSA, total surface area (cm^3^); ARD, average root diameter (mm); TRV, total root volume (cm^3^); TRT, total root tips; RF, root fork; SDW, shoot dry weight (mg plant^−1^); RDW, root dry weight (mg plant^−1^); TDW, total dry weight (mg plant^−1^); RSR, root to shoot ratio (mg mg^−1^); PupE, P uptake efficiency (mg plant^−1^); PutiE, P utilization efficiency (%); SP, sufficient P; LP, low P.

**Table 2:** Estimates of variance components and broad-sense heritability (H) for root and shoot traits under SP and LP conditions.

Trait	SP		LP		Combined Analysis		H
	Genotypes	H	Genotypes	H	Genotypes	Genotypes × P Levels	
PRL	170.06 **	0.83	95.83 **	0.75	185.89 **	80.69 **	0.77
TRL	245,586.94 **	0.87	283,624.31 **	0.81	373,387.29 **	155,823.95 **	0.68
TSA	5281.87 **	0.78	2798.48 **	0.77	5911.29 **	2169.04 **	0.63
ARD	0.0017 ^ns^	0.27	0.0021 **	0.56	0.0020 ^ns^	0.0018 ^ns^	0.51
TRV	0.44 **	0.68	0.28 **	0.86	0.51 **	0.22 **	0.58
TRT	106,729.27 **	0.77	55,261.34 **	0.75	94,974.36 **	67,016.26 **	0.60
RF	3,024,814.93 **	0.82	628,467.26 **	0.79	2,552,335.63 **	1,100,946.56 **	0.87
SDW	5824.96 **	0.64	1919.93 **	0.70	4001.09 **	3743.80 **	0.62
RDW	2130.70 **	0.75	295.78 **	0.76	1312.65 **	1113.83 **	0.55
TDW	13,929.02 **	0.78	3350.26 **	0.81	9057.42 **	8221.86 **	0.59
RSR	0.036 ^ns^	0.71	0.053 ^ns^	0.73	0.049 ^ns^	0.04 ^ns^	0.38
PupE	19,779.26 **	0.77	3278.75 **	0.83	12,490.84 **	10,567.17 **	0.66

SP, sufficient phosphorus; LP, low phosphorus; PRL, primary root length (cm); TRL, total root length (cm); TSA, total surface area (cm^3^); ARD, average root diameter (mm); TRV, total root volume (cm^3^); TRT, total root tips; RF, root fork; SDW, shoot dry weight (mg plant^−1^); RDW, root dry weight (mg plant^−1^); TDW, total dry weight (mg plant^−1^); RSR, root to shoot ratio (mg mg^−1^); PupE, P uptake efficiency (mg plant^−1^); PutiE, P utilization efficiency (%); ** significance at 0.01; ns, non-significant.

**Table 3:** Pearson correlation analysis for root and shoot traits under SP (upper diagonal) conditions and LP (lower diagonal) conditions.

Traits	PRL	TRL	TSA	ARD	TRV	TRT	RF	SDW	RDW	TDW	RSR	PupE
PRL	1	0.31 **	0.26 **	−0.05 ^ns^	0.29 **	0.22 **	0.26 **	0.04 ^ns^	0.02 ^ns^	0.02 ^ns^	0.12 ^ns^	0.17 ^ns^
TRL	0.39 **	1.00	0.87 **	0.01 ^ns^	0.84 **	0.71 **	0.69 **	−0.03 ^ns^	0.17 ^ns^	0.09 ^ns^	0.01 ^ns^	0.19 ^ns^
TSA	0.38 **	0.91 **	1.00	0.14 ^ns^	0.88 **	0.70 **	0.72 **	0.06 ^ns^	0.20 *	−0.07 ^ns^	−0.05 ^ns^	0.22 *
ARD	0.27 **	0.17 ^ns^	0.27 **	1.00	0.12 ^ns^	−0.03 ^ns^	0.12 ^ns^	0.08 ^ns^	−0.05 ^ns^	0.05 ^ns^	−0.10 ^ns^	−0.05 ^ns^
TRV	0.39 **	0.89 **	0.91 **	0.36 **	1.00	0.71 **	0.76 **	0.07 ^ns^	0.10 ^ns^	0.13 ^ns^	−0.04 ^ns^	0.24 *
TRT	0.35 **	0.75 **	0.74 **	0.17 ^ns^	0.61 **	1.00	0.55 **	0.13 ^ns^	0.13 ^ns^	0.14 ^ns^	0.009 ^ns^	0.18 ^ns^
RF	0.24 *	0.82 **	0.81 **	0.15 ^ns^	0.76 **	0.61 **	1.00	−0.18 ^ns^	−0.17 ^ns^	−0.14 ^ns^	0.05 ^ns^	0.20 ^ns^
SDW	0.16 ^ns^	0.17 ^ns^	0.25 *	−0.13 ^ns^	0.25 *	0.24 *	0.11 ^ns^	1.00	0.67 **	0.94 **	0.44 **	−0.16 ^ns^
RDW	−0.07 ^ns^	0.35 **	0.29 **	−0.05 ^ns^	0.32 **	0.29 **	0.37 **	0.70 **	1.00	0.89 **	0.12 ^ns^	0.26 *
TDW	−0.18 ^ns^	0.24 *	0.14 ^ns^	−0.08 ^ns^	0.28 *	0.22 *	−0.13 ^ns^	0.92 **	0.84 **	1.00	0.24 **	0.51 *
RSR	−0.16 ^ns^	−0.13 ^ns^	−0.18 ^ns^	−0.14 ^ns^	−0.18 ^ns^	−0.20 ^ns^	−0.20 ^ns^	−0.07 ^ns^	0.08 ^ns^	0.12 ^ns^	1.00	0.12 ^ns^
PupE	−0.12 ^ns^	0.37 **	0.32 **	−0.14 ^ns^	−0.11 ^ns^	0.28 **	0.17 ^ns^	0.48 **	0.41 **	0.50 **	−0.21 ^ns^	1.00

Significance * and ** significant at, *p <* 0.05 and *p <* 0.01, ns, non-significant. SP, sufficient phosphorus; LP, low phosphorus; PRL, primary root length (cm); TRL, total root length (cm); TSA, total surface area (cm^3^); ARD, average root diameter (mm); TRV, total root volume (cm^3^); TRT, total root tips; RF, root fork; SDW, shoot dry weight (mg plant^−1^); RDW, root dry weight (mg plant^−1^); TDW, total dry weight (mg plant^−1^); RSR, root to shoot ratio (mg mg^−1^); PupE, P uptake efficiency (mg plant^−1^).

**Table 4:** Selected lentil genotypes belonging to the top (8) 10% for TDW and SDW, along with the same genotypes also being in the top 10% for RDW, PupE and PUtiE.

Genotypes	TDW	SDW	RDW	PupE	PutiE
L4727	●	●			
EC718309	●	●	●	●	
EC714238	●	●	●		
PL-97	●	●			
EC718348	●	●	●	●	
DPL15	●		●		
PL06	●	●	●		●
EC718332	●	●		●	●

TDW, total dry weight (mg); SDW, shoot dry weight (mg); RDW, root dry weight (mg); PupE, P uptake efficiency (mg plant^−1^); and PutiE, P utilization efficiency (%).

**Table 5:** Analysis of variance (ANOVA) for tested root traits in identified genotypes under two P levels.

Source of Variation	TRL	TSA	TRV	TRT	RF
Genotypes (G)	236,318.26 **	1079.60 **	0.22 **	78,185.22 **	84,840.08 **
Phosphorus (P)	278,296.26 **	2570.80 **	0.18 **	106,408.33 **	227,395.75 **
G × P	44,739.47 **	407.51 **	0.04 **	16,310.52 **	34,350.79 **

TRL, total root length (cm); TSA, total surface area (cm^3^); ARD, average root diameter (mm); TRV, total root volume (cm^3^); TRT, total root tips; RF, root fork; ** significance at 0.01.

**Table 6:** Mean value of the studied traits of five genotypes selected based on PupE and PutiE under LP.

Genotypes	PRL	TRL	TSA	ARD	TRV	TRT	RF	SDW	RDW	TDW	RSR	PupE	PutiE
**PupE**													
EC718332	32.00	341.35	29.26	0.36	0.28	251.33	315.67	130.00	80.00	210.00	0.62	266.49	92.65
EC718348	39.33	85.60	10.11	0.41	0.10	188.33	47.67	130.00	86.67	216.67	0.67	263.25	59.09
EC718309	38.00	776.93	92.48	0.37	0.65	336.00	1718.33	140.00	83.33	223.33	0.60	257.65	82.72
EC718339	34.00	88.34	11.79	0.40	0.13	68.00	69.00	123.33	60.00	183.33	0.49	192.29	50.93
EC714238	18.67	393.43	45.53	0.38	0.33	269.33	324.33	136.67	83.33	220.00	0.61	189.62	60.55
**PutiE**													
IG69568	37.00	717.64	87.89	0.41	0.89	289.00	913.33	80.00	50.00	130.00	0.63	62.89	97.50
DPL62	42.33	834.75	112.29	0.42	1.12	484.33	826.00	133.33	73.33	206.67	0.55	119.81	96.88
SEHORE 74-3	48.00	325.25	48.15	0.37	0.52	301.33	299.00	113.33	83.33	196.67	0.74	59.42	96.72
MC6	38.67	705.33	77.63	0.37	0.67	427.00	427.00	126.67	80.00	206.67	0.63	74.98	95.38
PL06	35.00	513.27	71.66	0.42	0.81	375.33	485.00	130.00	80.00	210.00	0.62	115.07	94.03

PRL, primary root length (cm); TRL, total root length (cm); TSA, total surface area (cm^3^); ARD, average root diameter (mm); TRV, total root volume (cm^3^); TRT, total root tips; RF, root fork; SDW, shoot dry weight (mg); RDW, root dry weight (mg); TDW, total dry weight (mg); RSR, root to shoot ratio (mg mg^−1^); PupE, P uptake efficiency (mg plant^−1^); PutiE, P utilization efficiency (%); LP, low Phosphorus.

## Data Availability

Data is contained within the article or supplementary material.

## Supplementary Materials

The following are available online at https://www.mdpi.com/article/10.3390/plants10122711/s1. Table S1: *Lens* genotypes characterized for traits related to phosphorus use efficiency; Table S2: Classification of genotypes into efficient (E), medium (M) and inefficient (I) types based on five traits recorded under normal and low phosphorus conditions. Table S3: Phosphorus deficiency tolerance indices were calculated for 85 genotypes grown under high and low phosphorus conditions.
