# Supplementary material for: Genetic Variation for Traits Related to Phosphorus Use Efficiency in Lens Species at the Seedling Stage
Source: Plants (Basel). 2021 Dec 10;10(12):2711. doi: 10.3390/plants10122711 (PMC8707046; doi:10.3390/plants10122711)
Supplement: Supplementary file 1 [file plants-10-02711-s001.zip › plants-1459030-supplementary.pdf]

Table S1: *Lens* genotypes characterized for traits related to phosphorus use efficiency

Sl. No.	Species	Genotype(s)
1.	*Lens culinaris* subspp. *culinaris* Cultivated varieties	L4717, L4147, L4727, L4076, PL06, PL02, DPL62, K75, IPL81, IPL220, JL-1, IPL-321, L4594, DPL15, NDL1, K-75, WBL-77, IPL-316, HUL-57, SEHORE 74-3, PL-05, L-830, L4729, JL-7, HM-1
	Advanced breeding lines	L4618, BM4, KBL-104, LH84-8, LL931, L4603, PRECOZ, PL-97, PL117, KLS-218, MC6, L4649, IG69568, IC321808, IC560135, IG136607, I-G-Y-5, IG134340, LL699, IC268238, IPL7103, L 04610, IG129560, L4650, P43120, IG115, IC208352, L4602
2.	*L. ervoides*	Wild EC 718383, EC 718355, EC718351, EC718330, EC718339, EC718366, EC718347, EC718409, EC718329, EC718348, EC718332, EC718326, EC718331, EC718336
3.	*L.c. ssp. odemensis*	EC 718306, EC 718464, EC718283, EC718287, EC718309, EC718312, EC718243, EC718276, EC718677, EC718292, EC718297, EC718281, EC718295, EC718282
4.	*L. lamottei*	EC718692,
5.	*L. nigricans*	EC714238
6.	*L. orientalis*	ILWL-118

Table S 2: Classification of genotypes into efficient (E), medium (M) and inefficient (I) types based on five traits recorded under normal and low phosphorus conditions.

Genotypes	Low Phoshorus					High Phoshorus												
	SDW	RDW	TDW	RSR	P%	PupE	PutiE	Total score/ out of 20	SDW	RDW	TDW	RSR	P%	PupE	PutiE	Total score/ out of 20	Total score/ out of 40
L4717	M	M	M	M	E	M	M	15	M	M	M	I	M	M	M	13	28
L4618	M	M	M	M	M	M	M	14	M	M	M	M	M	M	M	14	28
L4147	E	M	M	I	M	E	E	16	M	M	M	M	M	M	E	15	31
L4727	E	M	E	I	M	M	M	15	E	M	M	I	M	M	M	14	29
L4076	M	M	M	M	M	M	M	14	M	I	M	I	M	M	M	12	26
BM4	E	M	E	I	M	M	E	16	M	M	M	M	M	M	E	15	31
PL06	E	E	E	M	M	M	E	18	M	M	M	I	M	M	E	14	32
PL02	M	M	M	M	M	M	E	15	I	I	I	M	M	M	E	12	27
DPL62	E	E	E	I	M	M	E	17	M	I	M	I	M	M	E	13	30
WBL-81	E	E	E	M	M	M	E	18	M	M	M	M	M	I	E	14	32
IPL81	E	M	E	M	M	M	M	16	M	E	M	E	M	M	M	16	32
IPL220	M	M	M	M	M	M	E	15	I	I	I	M	E	M	E	13	28
JL-1	I	I	I	M	E	M	M	12	M	M	M	M	M	M	M	14	26
IPL-321	M	M	M	M	M	M	I	13	E	M	E	I	I	M	I	13	26
KBL-104	M	M	M	M	M	M	M	14	M	M	M	M	M	M	M	14	28
L4594	M	M	M	M	M	M	M	14	M	M	M	M	M	M	M	14	28
LH84-8	M	I	M	I	M	M	E	13	I	M	I	E	E	M	E	15	28
LL931	M	E	E	M	E	M	M	17	E	M	E	M	I	I	M	14	31
DPL15	E	E	E	M	M	M	M	17	E	E	E	M	I	I	M	15	32
NDL1	M	E	M	E	M	M	M	16	M	M	M	M	M	I	M	13	29
K-75	M	M	M	E	E	M	M	16	M	M	M	M	M	M	M	14	30
WBL-77	I	M	M	E	E	M	M	15	I	M	I	E	E	E	M	15	30
IPL-316	M	M	M	M	E	M	M	15	M	M	M	M	M	M	M	14	29
L4603	M	M	M	M	M	M	M	14	M	M	M	M	M	M	M	14	28
PRECOZ	I	M	I	E	E	M	M	14	I	M	I	E	E	E	M	15	29
HUL-57	E	M	E	I	M	M	E	16	M	M	M	M	E	E	E	17	33
SEHORE 74-3	E	E	E	M	M	M	E	18	M	M	M	M	M	M	E	15	33
PL-97	E	E	E	I	I	M	E	16	M	M	M	M	M	E	E	16	32
IPL-406	M	M	M	M	I	M	I	12	M	E	E	E	M	E	I	17	29
PL-05	M	M	M	M	M	M	I	13	E	E	E	E	M	E	I	18	31
PL117	M	M	M	M	M	M	I	13	E	E	E	M	M	M	I	16	29
KLS-218	M	M	M	M	M	M	I	13	M	M	M	I	M	E	I	13	26
L-830	M	M	M	M	M	M	M	14	M	M	M	M	E	M	M	15	29
L4729	M	M	M	M	M	M	M	14	M	M	M	M	M	E	M	15	29
MC6	E	E	E	M	E	M	M	18	M	M	M	M	E	M	M	15	33
JL-7	M	M	M	M	M	M	E	15	I	I	I	M	M	M	E	12	27
HM-1	M	M	M	E	E	E	M	17	I	M	I	E	M	M	M	13	30
L4649	M	I	M	M	M	E	E	15	M	I	M	M	E	E	E	16	31

IG69568	M	M	M	M	M	E	M	15	I	I	I	M	E	M	M	12	27
IC321808	M	I	I	M	M	M	E	13	M	M	M	M	E	M	E	16	29
IC560135	M	M	M	M	E	M	I	14	M	M	M	M	M	M	M	14	28
IG136607	M	M	M	M	M	M	M	14	M	M	M	E	M	M	M	15	29
I-G-Y-50	M	E	M	M	M	I	M	14	M	I	M	I	M	M	M	12	26
IG134340	M	M	M	M	M	M	E	15	M	M	M	M	M	M	E	15	30
LL699	M	M	M	M	M	I	M	13	M	E	M	E	M	M	M	16	29
IC268238	M	M	M	M	M	M	M	14	E	E	E	M	M	M	M	17	31
IPL7103	M	M	M	M	M	M	I	13	E	M	E	M	M	E	I	16	29
L4610	I	M	M	M	M	M	I	12	M	M	M	M	M	M	I	13	25
IG129560	M	I	M	I	M	M	M	12	M	M	M	M	M	M	M	14	26
L4650	M	M	M	M	I	M	I	12	I	M	I	M	M	M	I	11	23
P43120	M	M	M	M	M	M	E	15	I	I	I	M	E	M	E	13	28
IG115	M	M	M	M	M	M	M	14	M	M	M	M	M	E	M	15	29
IC208352	M	M	M	M	M	M	M	14	M	E	M	E	M	M	M	16	30
L4602	M	M	M	M	I	M	M	13	E	E	E	M	M	M	M	17	30
EC718383	M	M	M	M	M	M	I	13	M	M	M	M	M	M	I	13	26
EC 718355	I	M	M	E	M	M	M	14	M	M	M	E	M	M	M	15	29
EC 718351	I	M	M	E	I	M	M	13	M	M	M	M	M	M	M	14	27
EC 718306	I	M	M	E	M	M	M	14	E	M	E	M	M	M	M	16	30
EC 718464	M	M	M	M	I	M	I	12	M	M	M	M	M	M	I	13	25
EC718292	M	M	M	M	M	M	I	13	I	M	I	E	M	M	I	12	25
EC718692	I	M	M	E	M	M	M	14	M	M	M	M	E	I	M	14	28
EC714238	E	E	E	M	E	M	E	19	E	E	E	M	M	M	E	18	37
EC718332	E	E	E	M	M	E	M	18	M	M	M	M	I	I	M	12	30
EC718283	M	M	M	M	M	E	E	16	M	M	M	M	I	I	E	13	29
EC718297	M	M	M	M	M	E	M	15	I	M	I	E	I	M	M	12	27
EC718339	E	M	E	I	M	E	M	16	E	E	E	M	E	M	M	18	34
EC718366	M	M	M	M	M	E	M	15	E	E	E	M	I	M	M	16	31
EC718347	M	M	M	E	M	M	I	14	M	M	M	M	M	M	I	13	27
EC718409	M	M	M	M	M	M	M	14	M	M	M	M	M	I	M	13	27
EC718329	M	M	M	M	I	M	M	13	M	M	M	M	M	M	M	14	27
EC718287	M	M	M	M	M	M	M	14	M	M	M	E	M	I	M	14	28
EC718348	E	E	E	M	I	M		14	E	E	E	M	I	M	M	16	30
EC718309	E	E	E	I	I	E	M	16	M	M	M	E	I	M	M	14	30

EC718330	M	I	M	M	I	E	E	14	M	M	M	M	M	M	E	15	29
EC718326	M	M	M	M	M	E	M	15	M	M	M	M	M	M	M	14	29
EC718312	M	M	M	M	I	M	I	12	E	E	E	M	M	M	I	16	28
EC718281	M	M	M	M	I	M	I	12	E	E	E	M	M	I	I	15	27
EC718295	M	M	M	M	M	M	I	13	E	E	E	M	I	I	I	14	27
EC718331	E	M	E	I	M	M	I	14	M	M	M	M	I	M	I	12	26
EC718243	M	E	M	E	I	M	M	15	M	M	M	I	M	M	M	13	28
EC718276	M	M	M	E	I	M	M	14	M	M	M	I	M	E	M	14	28
EC718282	I	M	M	E	I	I	M	12	M	M	M	M	E	I	M	14	26
EC718336	M	M	M	E	M	I	M	14	M	M	M	M	I	M	M	13	27
EC718677	E	M	E	I	M	I	M	14	M	M	M	M	M	I	M	13	27
ILWL-118	I	M	M	E	M	I	E	14	M	M	M	M	I	M	E	14	28

Table S3: Phosphorus deficiency tolerance indices were calculated for 85 genotypes grown under high and low phosphorus conditions.

Genotypes	Phosphorus Deficiency Tolerance Indices							
	SSI	MPI	GMPI	HMI	STI	TI	SI	STS
DPL15	1.20	100.53	2.88	2.91	3.37	1.43	-0.66	110.46
EC714238	0.97	79.56	2.45	2.66	2.75	0.57	-0.13	87.86
EC718348	1.00	79.56	2.43	2.61	2.72	0.66	-0.21	87.78
EC718281	1.70	73.97	1.38	0.69	1.37	2.75	-1.76	78.40
EC718295	1.50	69.78	1.64	1.29	1.69	1.98	-1.31	75.07
PL-05	1.59	66.98	1.41	0.92	1.40	2.25	-1.52	71.44
L4727	0.65	62.78	2.08	2.41	2.25	-0.24	0.57	69.85
EC718339	1.20	62.78	1.78	1.78	1.86	1.03	-0.65	68.58
EC718366	1.65	62.78	1.19	0.61	1.14	2.38	-1.65	66.45
LL931	1.15	57.19	1.66	1.69	1.71	0.85	-0.55	62.55
IC268238	1.60	53.00	1.01	0.52	0.94	2.07	-1.54	56.00
EC718312	1.53	46.01	0.93	0.55	0.85	1.75	-1.39	48.69
EC718309	0.42	41.81	1.49	1.82	1.50	-0.74	1.07	46.96
PL117	1.56	40.41	0.73	0.32	0.64	1.75	-1.45	42.40
L4602	1.52	39.02	0.75	0.39	0.66	1.61	-1.37	41.07
IPL81	0.93	36.22	1.17	1.32	1.12	0.17	-0.06	39.94
IPL-321	1.53	34.82	0.63	0.26	0.53	1.57	-1.38	36.43
PL-97	0.27	27.83	1.08	1.39	1.02	-1.01	1.41	31.72
IPL-406	1.49	23.64	0.37	0.07	0.27	1.30	-1.29	24.35
EC 718306	1.62	23.64	0.19	-0.28	0.09	1.66	-1.59	23.70
IPL7103	1.58	22.24	0.22	-0.21	0.11	1.52	-1.50	22.39
EC718332	0.18	18.04	0.78	1.08	0.69	-1.15	1.61	21.06
EC718331	0.50	18.04	0.75	1.01	0.65	-0.69	0.89	20.65
PL06	0.15	16.65	0.74	1.03	0.65	-1.19	1.68	19.56
EC718326	1.39	15.25	0.25	0.05	0.14	0.94	-1.06	15.56
MC6	0.11	12.45	0.61	0.90	0.51	-1.24	1.76	14.99
DPL62	0.08	11.05	0.57	0.85	0.47	-1.28	1.84	13.50
EC 718464	1.40	12.45	0.15	-0.06	0.05	0.94	-1.10	12.43
WBL-81	0.26	9.66	0.52	0.79	0.41	-1.06	1.43	11.75
IG129560	1.39	11.05	0.13	-0.08	0.02	0.89	-1.07	10.95
BM4	0.40	6.86	0.42	0.67	0.31	-0.88	1.12	8.50
EC718287	1.04	6.86	0.25	0.30	0.14	0.12	-0.29	7.38
IC208352	1.35	6.86	0.04	-0.13	-0.06	0.75	-0.99	6.48
SEHORE 74-3	0.08	2.67	0.31	0.57	0.20	-1.28	1.83	4.30
EC718677	0.23	2.66	0.31	0.56	0.20	-1.10	1.49	4.12
HUL-57	0.20	1.27	0.27	0.51	0.16	-1.15	1.57	2.63
LL699	1.31	2.67	-0.05	-0.19	-0.14	0.62	-0.90	2.01
EC718330	1.36	2.66	-0.08	-0.26	-0.17	0.71	-1.00	1.87
EC718383	0.99	-0.13	0.06	0.12	-0.04	-0.01	-0.19	-0.19
L4717	1.19	-0.13	-0.04	-0.10	-0.14	0.35	-0.63	-0.69
EC718243	0.66	-2.93	0.08	0.25	-0.02	-0.56	0.55	-2.62
KBL-104	1.11	-2.93	-0.08	-0.08	-0.17	0.17	-0.45	-3.54

EC718276	1.11	-2.93	-0.08	-0.08	-0.17	0.17	-0.45	-3.54
NDL1	0.83	-7.12	-0.09	0.02	-0.18	-0.33	0.17	-7.52
IG136607	1.11	-7.12	-0.20	-0.21	-0.28	0.12	-0.44	-8.13
L4147	0.40	-8.52	-0.05	0.15	-0.14	-0.92	1.12	-8.36
EC718347	1.17	-9.92	-0.32	-0.37	-0.38	0.21	-0.59	-11.36
KLS-218	0.85	-12.72	-0.27	-0.17	-0.34	-0.33	0.12	-13.70
EC718283	0.85	-12.72	-0.27	-0.17	-0.34	-0.33	0.12	-13.70
IPL-316	0.91	-12.71	-0.29	-0.21	-0.35	-0.24	-0.01	-13.82
I-G-Y-50	0.41	-14.11	-0.22	-0.04	-0.30	-0.92	1.09	-14.50
L4603	0.95	-14.11	-0.34	-0.29	-0.40	-0.20	-0.10	-15.43
L4594	1.06	-14.11	-0.39	-0.38	-0.44	-0.01	-0.35	-15.68
EC 718355	1.27	-14.11	-0.50	-0.62	-0.54	0.35	-0.81	-16.23
IC560135	1.21	-15.51	-0.50	-0.58	-0.54	0.21	-0.66	-17.57
L4618	1.00	-18.31	-0.48	-0.46	-0.52	-0.15	-0.21	-20.13
L-830	0.79	-19.71	-0.46	-0.36	-0.50	-0.47	0.26	-21.23
EC718329	0.92	-19.71	-0.50	-0.44	-0.53	-0.29	-0.02	-21.49
IG115	1.04	-19.70	-0.54	-0.53	-0.57	-0.11	-0.29	-21.74
EC718336	0.93	-22.50	-0.58	-0.54	-0.61	-0.29	-0.06	-24.57
K-75	0.99	-22.50	-0.61	-0.58	-0.62	-0.20	-0.19	-24.70
EC 718351	1.05	-26.70	-0.75	-0.76	-0.74	-0.15	-0.31	-29.41
IG134340	1.03	-28.09	-0.78	-0.78	-0.76	-0.20	-0.26	-30.89
L4729	1.07	-29.49	-0.84	-0.86	-0.81	-0.15	-0.35	-32.51
EC718409	0.91	-30.89	-0.83	-0.79	-0.80	-0.38	-0.01	-33.70
L4610	1.08	-32.29	-0.93	-0.96	-0.88	-0.15	-0.39	-35.60
L4076	0.47	-33.69	-0.83	-0.70	-0.80	-0.92	0.97	-35.97
EC718692	0.94	-36.48	-1.01	-0.99	-0.93	-0.38	-0.08	-39.87
EC718282	0.94	-36.48	-1.01	-0.99	-0.93	-0.38	-0.08	-39.87
ILWL-118	0.94	-36.48	-1.01	-0.99	-0.93	-0.38	-0.08	-39.87
HM-1	0.31	-39.28	-0.99	-0.85	-0.92	-1.10	1.33	-41.81
L4649	0.86	-40.68	-1.11	-1.07	-1.01	-0.51	0.10	-44.28
IC321808	1.00	-40.68	-1.15	-1.16	-1.04	-0.33	-0.22	-44.58
PL02	0.21	-42.08	-1.07	-0.94	-0.98	-1.19	1.54	-44.72
EC718292	0.67	-42.08	-1.11	-1.03	-1.01	-0.74	0.52	-45.45
IPL220	0.27	-43.47	-1.11	-0.99	-1.01	-1.15	1.42	-46.31
L4650	0.37	-46.27	-1.21	-1.10	-1.08	-1.06	1.18	-49.52
EC718297	0.65	-46.27	-1.24	-1.16	-1.10	-0.78	0.57	-49.98
LH84-8	0.33	-47.67	-1.25	-1.14	-1.10	-1.10	1.29	-50.97
PRECOZ	0.86	-47.67	-1.32	-1.30	-1.15	-0.56	0.09	-51.90
JL-1	1.29	-47.67	-1.47	-1.62	-1.26	-0.01	-0.84	-52.88
WBL-77	0.75	-49.07	-1.34	-1.29	-1.17	-0.69	0.35	-53.21
JL-7	0.54	-53.26	-1.44	-1.36	-1.23	-0.92	0.80	-57.41
IG69568	0.06	-54.66	-1.45	-1.35	-1.24	-1.33	1.87	-58.16
P43120	0.56	-56.06	-1.52	-1.46	-1.29	-0.92	0.78	-60.47
